# Supplementary material for: Agonism of Prostaglandin E2 Receptor 4 Ameliorates Tubulointerstitial Injury in Nephrotoxic Serum Nephritis in Mice
Source: J Clin Med. 2021 Feb 18;10(4):832. doi: 10.3390/jcm10040832 (PMC7922874; doi:10.3390/jcm10040832)
Supplement: Supplementary file 1 [file jcm-10-00832-s001.pdf]

Supplementary Materials

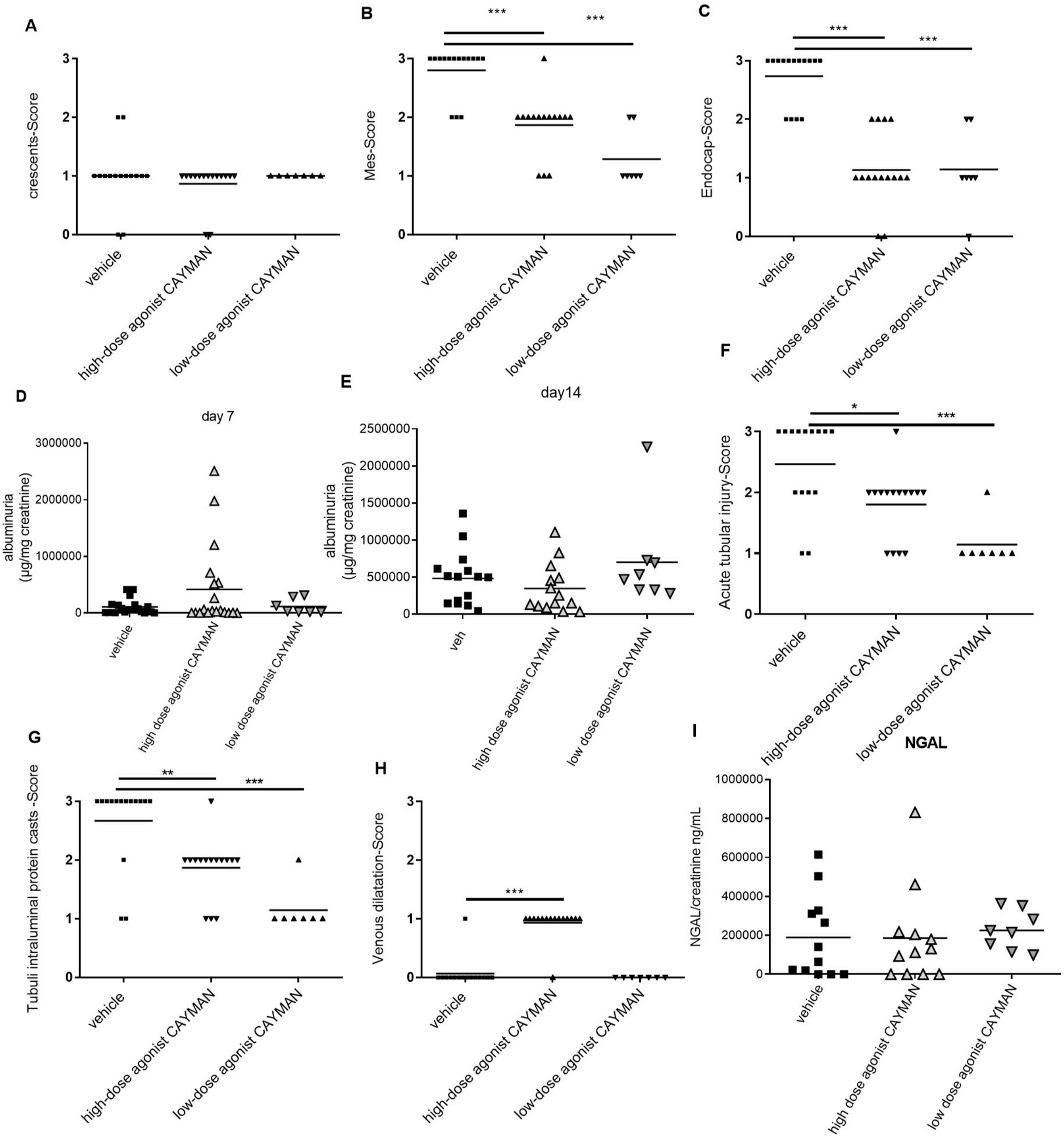

Figure S1. EP4 receptor agonism improves the phenotype of NTS. Fourteen days after NTS induction kidneys of mice treated with vehicle, high-dose EP4 receptor agonist (25 µg/mouse/day) or low-dose EP4 receptor agonist (7 µg/mouse/day; Cayman Chemicals, L-902,688) were harvested and stained kidney sections were quantified for glomerular and kidney damage (**A**–**C**). Urine samples collected on day 7 (**D**) and 14 (**E**) were analyzed for albumin and creatinine. In order to quantify for tubular damage acute tubular injury score (**F**) and tubular cast formation per 6 high power field (HPF) (**G**) were evaluated. Venous dilatation score (**H**) was quantified and serum NGAL levels (**I**) were evaluated on day 14. (*$p \leq 0.05$, **$p \leq 0.01$, ***$p \leq 0.001$).

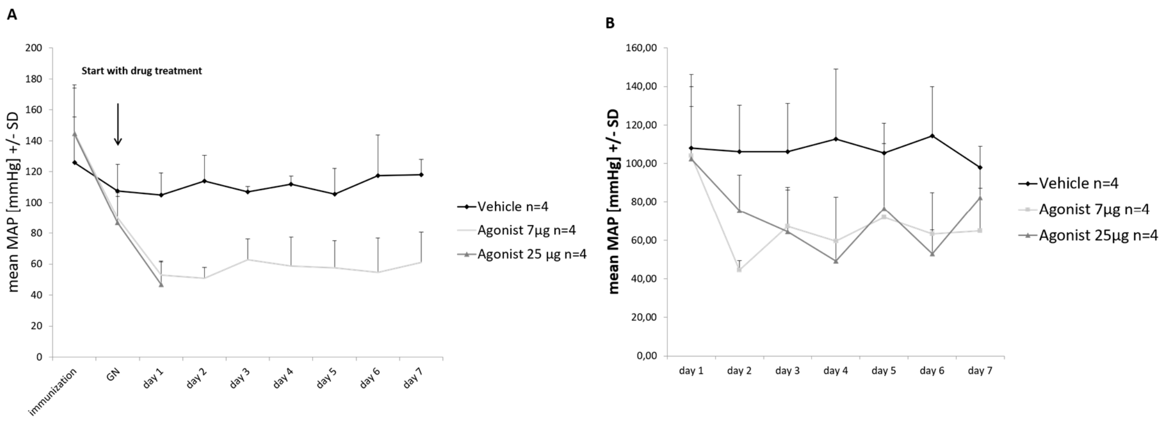

Figure S2. Hypotensive episodes occur after EP4 receptor agonist (Cayman Chemicals, L-902,688) administration. Blood pressure was assessed in vehicle (n = 4), EP4 receptor high-dose agonist (n = 4) and EP4 receptor low-dose agonist (n = 4) treated mice after NTS induction. Blood pressure was measured via the tail cuff method immediately (**A**) and 30 min after EP4 agonist injection (**B**) in all 3 groups. Mean arterial pressure (MAP) is represented as mean ± SEM.

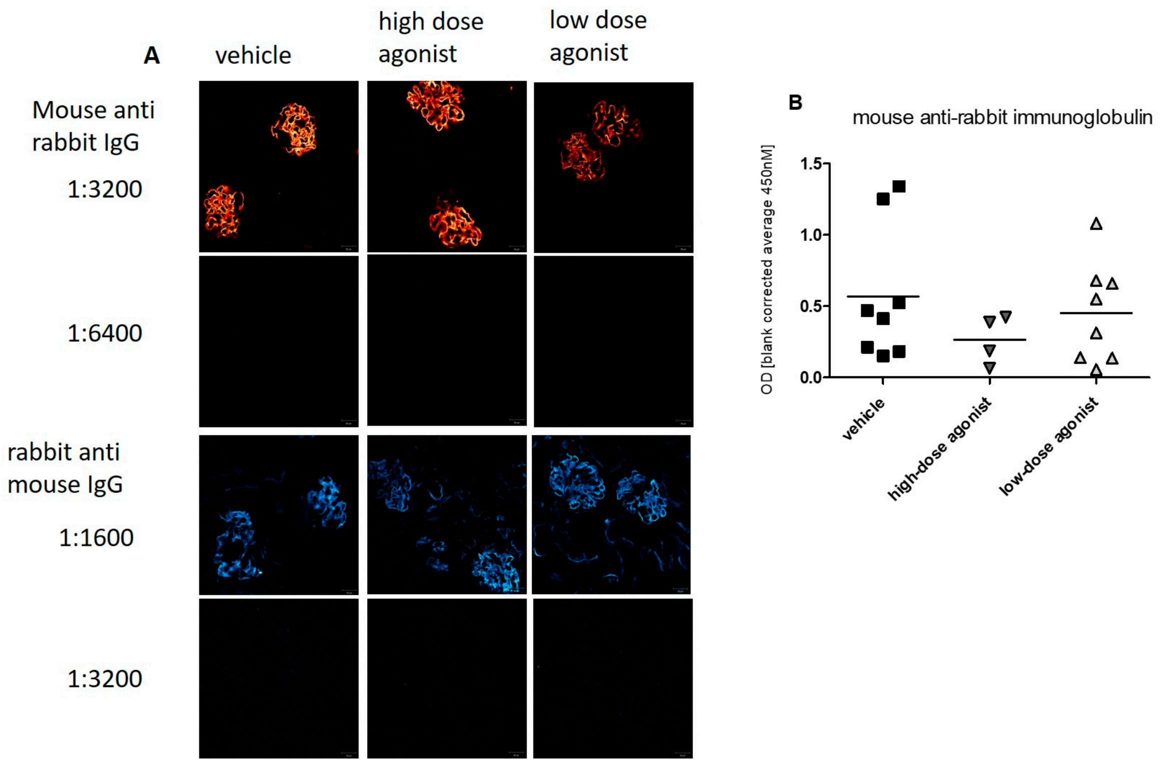

Figure S3. Treatment of NTS mice with EP4 antagonist or agonist does not affect IgG deposition in the kidney. Mouse anti-rabbit IgG serum titers were evaluated on day 14 after NTS induction (**A**). Furthermore, deposition of mouse anti-rabbit IgG and rabbit anti-mouse IgG on glomerular basal membrane was performed (**B**).

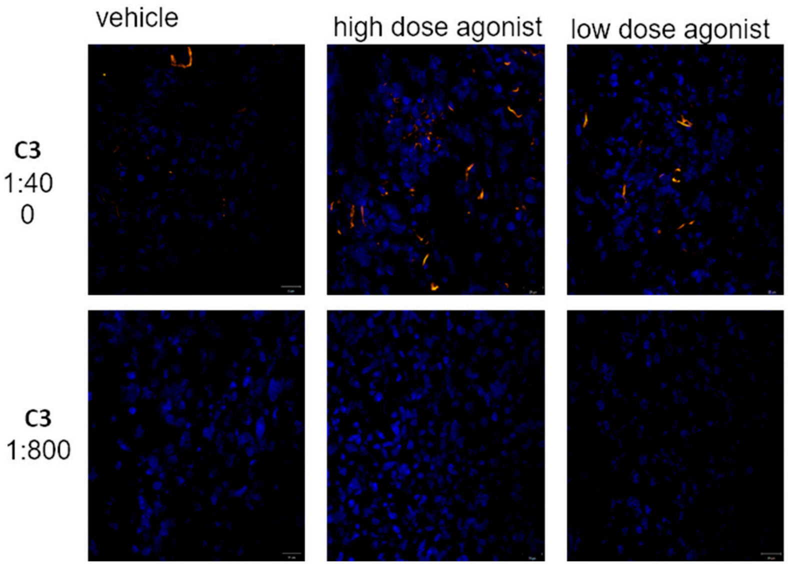

Figure S4. Treatment of NTS mice with EP4 agonist does not affect C3 deposition in the kidney. On day 14 after NTS induction C3 deposition was evaluated by immunofluorescence microscopy;.

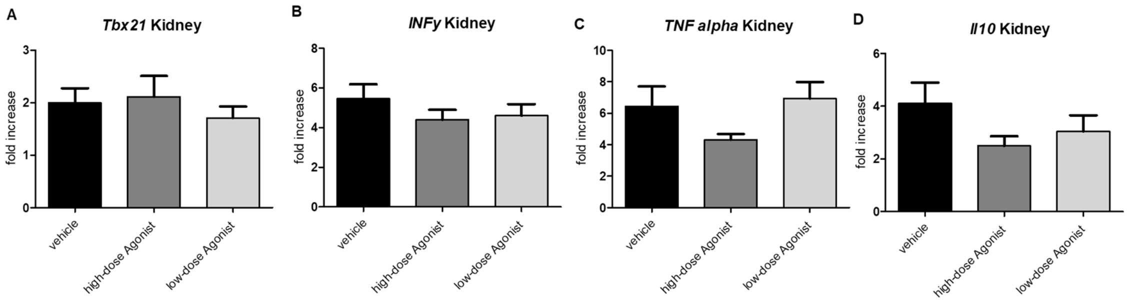

Figure S5. Cytokine mRNA expression in the kidney. Fourteen days after NTS induction RT PCR was performed to evaluate cytokine expression in the kidney (**A–D**) in mice treated with vehicle (*n* = 6), high-dose EP4 receptor agonist (*n* = 3-4), low-dose EP4 receptor agonist (*n* = 7-8) and compared to healthy mice kidneys (*n* = 3).

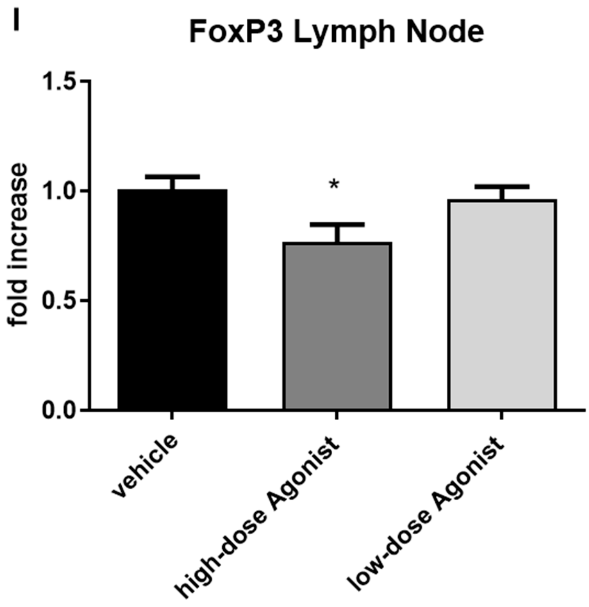

Figure S6. Proliferation of tubular cells in vivo. (A) Fourteen days after the induction of NTS mice treated with a high- (25 µg/mouse/day; *n* = 7) or low-dose (7 µg/mouse /day; *n* = 6) of the EP4 receptor

agonist (Cayman Chemicals, L-902,688) or vehicle ($n = 9$) were evaluated for the number of PCNA$^+$ tubular cells. *$p < 0.05$.

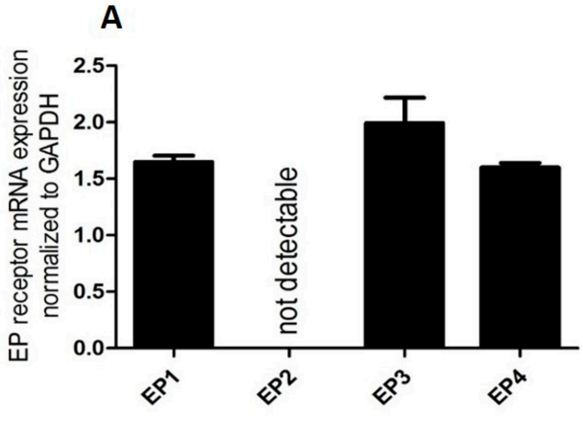

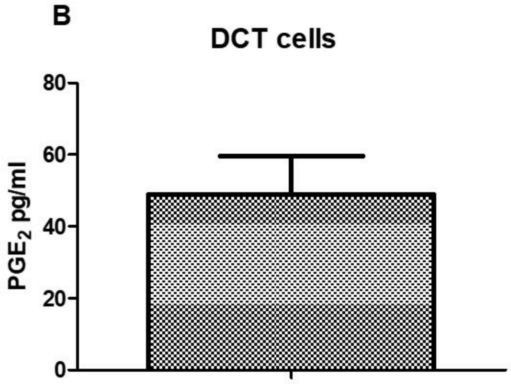

Figure S7. EP4 is expressed by Distal Convoluted Tubular cells. EP1, 2, 3 and 4 receptor mRNA expression was evaluated on DCT cells (**A**). PGE2 production of DCT cells after 72 h of incubation in starving medium was evaluated by RIA (48.9 ± 10.6 pg/mL) (**B**). Experiments were performed in duplicates and in total at least five different experiments were performed.
